# Landscape Perception and the Importance of Recreation Areas for Students during the Pandemic Time

**DOI:** 10.3390/ijerph19169837

**Published:** 2022-08-10

**Authors:** Sebastian Bernat, Karolina Trykacz, Jakub Skibiński

**Affiliations:** Institute of Socio-Economic Geography and Spatial Management, Maria Curie-Skłodowska University, Kraśnicka Av. 2d, 20-718 Lublin, Poland

**Keywords:** COVID-19, landscape, green space, recreation, well-being, young people

## Abstract

The landscape and its perception influence the quality of life of a local community. Recreation areas bring a lot of benefits to society, also in terms of mental health. Open space and contact with nature, particularly during a crisis such as the pandemic, help alleviate the effects of the lockdown and social isolation. The study objective was to determine whether and to what extent the COVID-19 pandemic influenced the importance of recreation areas and the perception of landscape among students—a social group that experiences mood disorders increasingly often and has been severely affected by the lockdown. A survey was conducted in two stages, using a Google Forms online survey. A total of 381 students from universities in Lublin participated in the survey. The survey showed that the significance of recreation areas increased during the pandemic. The perception of landscape changed as well: the value of nature, scenic views, and the therapeutic effect of the landscape began to be appreciated to a greater extent. The survey results indicate the need to ensure the diversity of green areas and improve their accessibility. Designing these areas should also consider quiet areas as well as ensuring green mobility and places of recreation.

## 1. Introduction

The SARS-CoV-2 virus causing COVID-19 emerged in December 2019 and quickly spread throughout the world. It caused a high morbidity and mortality [1]. Minimally, 486,761,597 cases of infection and over 6 million deaths resulting from the coronavirus have been confirmed by 1 April 2022. In Poland, nearly 6 million cases of infection and over 100 thousand deaths have been confirmed [2].

According to the relevant typology, pandemics are ranked among natural disasters, causing huge problems in terms of mental health. The SARS-CoV-2 virus has had a major impact on the mental health of societies, manifested in post-traumatic stress disorder both in persons that have had COVID-19 and those who have not contracted the coronavirus (also among young adults). It has also influenced a whole range of people’s behaviours and activities [1,3] and, consequently, the perception and development of space. Solutions aimed at social isolation, i.e., the lockdown measures (e.g., social distancing, ban on gatherings) and large-scale vaccination, have not led to the disappearance of the threat. After nearly three years, the coronavirus continues to mutate and control the life of societies, and it is difficult to estimate when the situation will become stable.

The COVID-19 pandemic is a global health crisis [4] that has also resulted in a crisis of the tourism industry due to travel restrictions [5]. The sense of safety and citizens’ rights to active leisure were violated on an unprecedented scale despite it being a time of peace [6]. The forced isolation impeded all kinds of human activity, including those in recreation areas [7]. The isolation has also impacted the mental health of people. Particularly in the initial phases of the lockdown, when access to recreation areas was restricted, thus reducing the level of daily activity [8], symptoms of depression were increasingly visible [9]. The amount of time spent by people in open space was clearly reduced, particularly in cities [10]. This is a reason for concern because outdoor activity plays a crucial role in the prevention of health issues [11,12].

Diverse activity undertaken in green areas can help solve problems related not only to physical but also mental health, because such areas offer possibilities to reduce stress levels and are conducive to social interaction. Thus, they foster well-being and mental health [13]. Contact with nature, particularly in stressful circumstances, can act as a kind of buffer protecting individuals against stress [9].

The role of public recreation areas in promoting a healthy lifestyle and restoring the physical and mental health of city dwellers was limited during the crisis caused by COVID-19 [14,15]. The response of state governments to the COVID-19 pandemic (lockdown) significantly decreased daily activity levels and restricted access to recreational areas. Despite numerous studies on the importance of recreation areas during the pandemic [7,8,9,10], particularly at its beginning, there is a gap in research linking the significance of recreation areas before and during the pandemic with landscape elements that influence the choice of recreation area, particularly from the students’ perspectives. Most of the studies have involved the general population, referenced later in the article. Understanding how a vulnerable population, such as students, has been affected by the pandemic, is a significant added value. University students were selected for the survey because they are a social group that is increasingly often diagnosed with mood disorders. This was emphasised, for example, by Szczepańska and Pietrzyk [16] who analysed well-being in young adults—students, and referenced research conducted by Białkowska et al., 2014 and Jaworska et al., 2014, among others. Their studies demonstrated that women suffered greater mood-related issues, and more than 10 percent of all the respondents experienced episodes of depression [17], while nearly 20% had mood-related issues [18]. According to Szczepańska and Pietrzyk’s study, for the majority (72%) of students, the state of the epidemic threat had a negative or rather negative impact on their daily activities in the public space [16]. Hence, research dedicated to this social group in the context of landscape and recreational areas before and during the pandemic seems justified.

The study objective was to determine the impact of the COVID-19 pandemic on the perception and preferences of landscape and importance of recreation areas and factors that influence their use. It used the example of students from universities in Lublin, a city in south-eastern Poland, located about 100 km from the border of the European Union. Landscape perception is defined as a process of a multi-sensory, emotional, and intellectual perception of landscape as a common good [19]. Identifying landscape preferences also appears as a significant part of landscape perception research. Furthermore, studying the changing perceptions of landscapes during the pandemic is important for the future sustainable development of landscape [20]. The study would also have a practical dimension, related to spatial planning, considering the accessibility of recreational areas as well as the design of quiet areas.

The survey was expected to answer the following research questions:

1. What are the students’ preferred landscapes for recreation purposes?
2. Did the COVID-19 pandemic result in a change in the perception of landscape? What did that change consist of?
3. How to design and develop the landscape to provide a richer experience, conducive to relaxation?
4. Were recreation areas conducive to improvement in well-being and stress reduction in students during the COVID-19 pandemic?
5. Did the opinions about the accessibility of recreation areas change during the COVID-19 pandemic?
6. Did the COVID-19 pandemic increase the frequency of the students’ use of recreation areas and what were the recreational activities practiced?
7. What types of recreation areas were chosen for leisure activity and what landscape features influenced the choice of a place for recreation and what were the most frequented specific places?

## 2. The Impact of Landscape and Recreational Areas on Health—A Literature Review

The European Landscape Convention [19] defined landscape as “an area, as perceived by people, whose character is the result of the action and interaction of natural and/or human factors”; it is regarded as a key element of the welfare of the entire society and individuals, and its preservation and planning entail certain duties for every person. Recreation areas are related to the appearance of certain elements and types of landscape. Recreational landscapes, used for recreation, used under the influence of recreation, with high attractiveness, suitability and recreational potential, with numerous tourist values, properly and attractively developed, are also distinguished [21,22]. Recreation and leisure areas are free from buildings and related facilities; they include areas of designed parks and green squares as well as non-designed green areas. They also include botanical and zoological gardens, areas with entertainment functions (e.g., amusement parks), various sports grounds, family-owned allotment gardens, areas of holiday centres, or areas of a historic nature [23]. Publicly accessible forests or boulevards [24], water reservoirs and their surroundings, and riverside areas also perform a recreation and leisure function [25,26]. These are both public and privately owned areas [27] that can be used for walks, hiking, running, cycling, camping, fishing, birdwatching and other recreation activities.

The influence of landscape and recreation areas on human health and quality of life has been recognised at least since the 1980s when Ulrich studies [28] have indicated that a view from the window was conducive to the recovery of hospital patients. In the 1990s, Gesler [29] put forward the concept of therapeutic landscapes, described by him as those changing places, settings, locales and milieus that encompass both the physical and psychological (non-tangible) environments associated with treatment or healing, helping achieve physical, mental and spiritual healing. Therapeutic landscapes are places of everyday health promotion, conducive to the restoration of physical and mental health, e.g., through contact with nature and possibility of experiencing it with various senses. Therapeutic places, a concept linked with therapeutic landscapes, are places with an enduring reputation for supporting the healing process [30]. These are, among others, health resorts, mountain and seaside areas, pilgrimage centres. These places are usually accompanied by valuable natural assets (mineral springs, forests), attractive landscapes, and, in the case of religious sites, an atmosphere of mysticism [31,32,33]. The idea of therapeutic landscapes was developed by Williams [34,35] who recognised spirituality as the most intangible dimension of these landscapes. The subject of the impact of landscape on health and quality of life has also been studied by scholars such as Liamputtong and Suwankhong [36], Rose [37], Houghton and Houghton [38], Bell et al. [39], Meijering et al. [40], Doughty [41], Tsunetsugu et al. [42], Thompson [43], and Abraham et al. [44]. This is related to the wide range of positive changes in brain activity, blood pressure, heart action, and muscle tension. As demonstrated by Wolf and Flora [45], the therapeutic role of a landscape is manifested in the improved mental state in persons affected by various mental conditions, including depression, from which, according to the WHO, about 350 million people currently suffer. According to Velarde et al. [46], natural landscapes and open vistas generally have a more positive impact on health as compared to urban, closed landscapes: they improve well-being, alleviate anxiety and pain, and reduce stress, blood pressure, heart rate, and muscle tension. An important therapeutic role is played by vegetation (including vegetation seen from the window and within a one-kilometre radius from one’s place of residence) and water. It is necessary to search for functional landscape models conducive to human health and sustainable development. Using the survey findings in the planning and designing of landscape is a key research challenge for the future. Menatti and de Rocha [47] observed that perception is the key to understanding the health–landscape relationship. It is through perception that we establish relationships with landscape which influences our health in its mental (intellectual, emotional), physical, social, and spiritual dimension.

Recreation areas, particularly in cities, bring various benefits, including ecological and social ones [15]. Access to natural areas and staying outdoors are often associated with mental well-being, which is also confirmed by numerous scientific studies, e.g., Refs. [9,48,49,50]. Exposure to green areas contributes to a reduction in diastolic blood pressure, salivary cortisol, and heart rate, diabetes incidence, stroke rates, overall mortality, and cardiovascular mortality, etc. An increased frequency of a high self-reported health status [51,52] can also be observed. Contact with nature has a positive influence on both physical and mental health. It has a reinvigorating effect thanks to the possibility of getting away from everyday matters and problems. It can also act as a kind of buffer protecting against stress in the face of difficult, stressful events or circumstances. It also provides “immunity” against stress that may occur in the future [9,53,54]. A positive influence of nature and “health-promoting landscapes” on concentration is also visible [43,54,55]. The soundscapes of city parks, often described as quiet areas, also play a considerable role in stress alleviation [56,57].

Quiet areas can be defined as those that have a pleasant soundscape, consisting of sounds of nature or those created by humans [58]. In Poland, ‘quiet areas’ as defined in the Act of 27 April 2001, The Environmental Protection Law [59], is divided in accordance with Directive 2002/49/EC (“Noise Directive”), into quiet areas within the agglomeration and quiet areas outside the agglomeration. The first are areas within which the allowed noise levels, as defined by the noise indicator L_DwN, are not exceeded. Quiet areas outside the agglomeration, are defined as those that are free of traffic, industrial or recreational noise ([59] art. 3). Indeed, the indication of quiet areas is binding for spatial planning instruments ([59] art. 73).

The delimitation of these areas in European cities is still underway. These areas must meet certain requirements. The criteria for the delimitation the areas in open spaces and the areas in urban areas are different. Following a study by the European Environment Agency in European countries, it transpired that 60% of those cities surveyed had designated at least one quiet zone [60]. It is worth emphasizing that in Sweden, the delimitation of quiet areas took place in the 1990s [61]. Furthermore, in the Netherlands (in Amsterdam), quiet areas are created in city parks, squares or resting places with low traffic volumes [62]. In Poland, it has been limited to proposing quiet areas in some cities (e.g., Gdynia, Radom). However, such areas have not been designated.

Natural areas thus constitute a major health-promoting resource [48]. The relevance of research on the impact of landscape on health is evidenced by new publications on the attributes of landscape that support health [63,64]. Studies in the context of COVID-19 are also being carried out. Doughty et al. paid attention to the landscape-health relationship that was linked to the pandemic period [65], whereas Liu et al. showed the influence of the landscape outside the window on COVID19 anxiety levels. The greatest satisfaction was with the waterscape and greenery [66]. Noszczyk et al. (2022) in their research considered the reasons for visiting green spaces during the pandemic period and identified the areas of green space that were most frequently chosen in Kraków. The improvement of general well-being was the most significant factor [67].

Besides bringing physical and mental health benefits, green areas are also a place of social interactions. They are conducive to undertaking various activities and strengthening the sense of community [13,68]. Furthermore, people who have access to green areas are less affected by loneliness [52]. Health-promoting landscapes are also conducive to the development of social support [44]. Green areas are also important in the context of safety. More verdure in urban areas leads to a reduction in aggressive behaviours [69]. To fully support the development of community ties, recreation areas, including green areas, must be properly maintained and equipped with various facilities [70]. Therefore, what is important is not only the existence of such areas but also their quality [68]. The way in which green areas are developed is very important for the sense of safety of their users just like it is for social interactions [13].

Crowded and indoor spaces are conducive to the transport of aerosols [71]. Due to the closing down of many facilities and services during the lockdown, many people began to miss physical activity, hence they started to look for alternatives. Outdoor recreation areas made it possible to engage in various forms of activity [15]. The pandemic thus led to an increased importance of recreation areas [50]. This was not only due to the fact that regular air flow disperses and dilutes contagious respiratory aerosols. Furthermore, contact with nature helps people cope with depression and anxiety resulting from the restrictions of the lockdown period [9]. Therefore, outdoor spaces play a very important role in combating the negative effects of social isolation. This has been confirmed by research conducted, e.g., by Lopez et al. [72] who showed that recreation areas during the pandemic were regarded by the respondents as “extremely” or “very important both for mental and physical health. It even seems that they were more crucial for mental well-being. The results of research by Poortinga et al. [27] indicate that subjectively improved well-being and self-reported health status were influenced not only by public green areas but also private spaces, including home gardens. Their importance increases with the growing distance from public areas.

During the pandemic, inhabitants choose the less crowded parts of the city for recreation [15]. In New York City, according to research by Lopes et al., walking lanes, trees, and shading were the key determinants influencing the choice of a recreation area [72]. International studies conducted by Ugolini et al. indicate that city parks were the most frequented places during the pandemic even though the frequency of these visits was smaller than before the pandemic. Changes in people’s motivation to take advantage of green areas could be observed. There was a greater need for “indispensable activities”, such as taking the dog for a walk, while non-essential or high-risk activities were limited [7]. Due the COVID-19 pandemic, many city dwellers in Poland started to buy properties in the suburbs or rural areas [15].

All social groups were affected by the spatial effects of the pandemic, which were identified in the case of senior citizens [73,74] and students [16,75], among other groups. They were observed in Italy [7,76], Spain [7,77], Croatia, Israel, Lithuania, Slovenia [7] and Poland [16,75], Ukraine and Belarus [75]. Surveys conducted in April 2020 (during the lockdown in Poland) reveal that students were strongly affected by the limited access to public space and deficiency of direct social interaction [16]. As in other countries, physical space was completely replaced by the virtual world and online communities [78]. However, online communication cannot fully compensate for the lack of direct contact with peers in a public space. Thus, the respondents showed a greater appreciation of unlimited everyday access to public space as having an influence on physical and mental health.

## 3. Study Area

The survey was conducted among students from universities in Lublin, the largest city in eastern Poland, a county seat and capital of Lubelskie Province. Lublin covers an area of 148 km^2^. A city with a centuries-old academic tradition, Lublin offers a wide range of education possibilities [79].

According to the physical-geographical division of Poland by Solona et al. [80], Lublin lies in the Lublin Upland macro-region, at the junction of three meso-regions: Bełżyce Plain, Nałęczów Plateau and Świdnik Plateau. The loess-covered Nałęczów Plateau features numerous erosion-denudation valleys and gullies The Bystrzyca and Wieprz rivers flow across the Świdnik Plateau. The Zemborzycki Lake built on the Bystrzyca in the 1970s function both as a water retention reservoir but also as a place of recreation for Lublin’s inhabitants [81].

**Figure 1:**
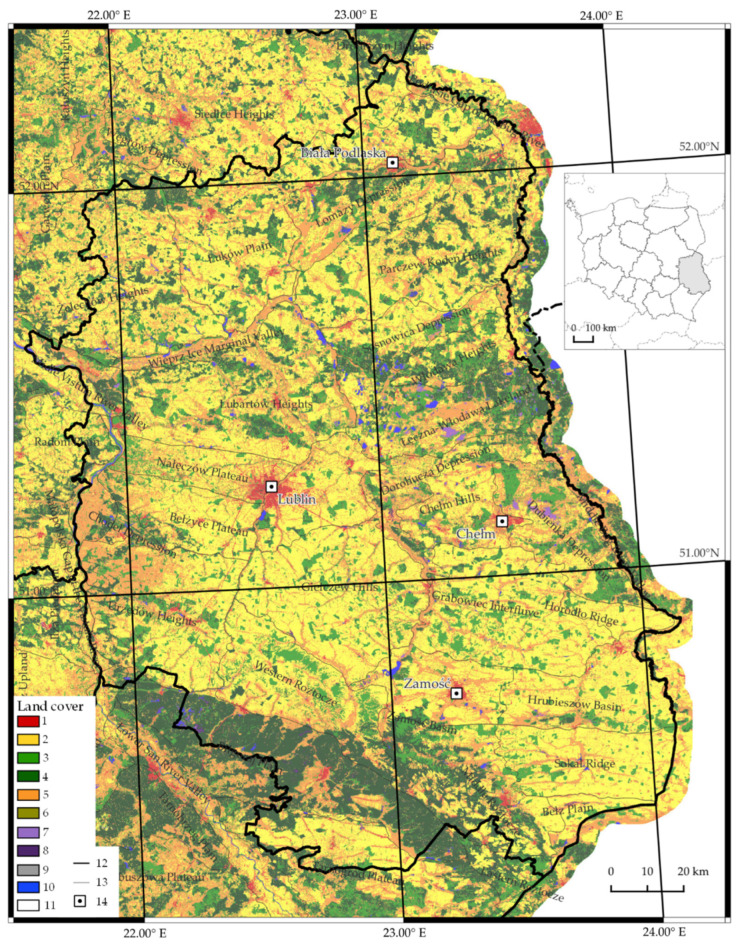
Land cover in Lubelskie Province against the physical-geographical division. (1) anthropogenic areas; (2) agricultural areas; (3) deciduous forests; (4) coniferous forests; (5) grassland; (6) heaths and shrubs; (7) wetlands; (8) peat-bogs; (9) natural areas devoid of vegetation; (10) water areas; (11) no data; (12) provinces; (13) mesoregions; (14) major towns and cities (source: [80,84]).

The meso-regions mentioned above border with the Lubartów High Plain and the Łęczna-Włodawa Lake District. Lake Firley, located in the fossil valley of the Wieprz river, within the Lubartów High Plain, is also a place of leisure and recreation for the inhabitants of Lublin, 40 km away. The nearby Kozłowieckie Forest is also a valuable resource [82]. The flat Łęczna-Włodawa Lake District comprises peatlands and wetlands (Figure 1). alongside 61 lakes and various forms of nature conservation: Poleski National Park, 3 landscape parks, 8 nature reserves, and 16 Natura 2000 areas. The lake district is a recreation area for the inhabitants of Lublin and the entire province [83].

In 2020, Lublin had a population of 338,856. Young adults (aged 19–24) accounted for about 5% of the inhabitants. In terms of the number of students, Lublin is among the top academic centres in Poland and the largest in the eastern part of the country. There are nine universities (five public and four non-public) with about 60,000 students, including about 7000 foreign students [79,85]. According to the global Ranking Web of Universities, the highest rank among the universities in Lublin was awarded to the Lublin University of Technology (18th in Poland and 1400th in the world), followed by Maria Curie-Skłodowska University (21st and 1617th respectively) and the Medical University (33rd and 2202nd respectively) [86].

**Figure 2:**
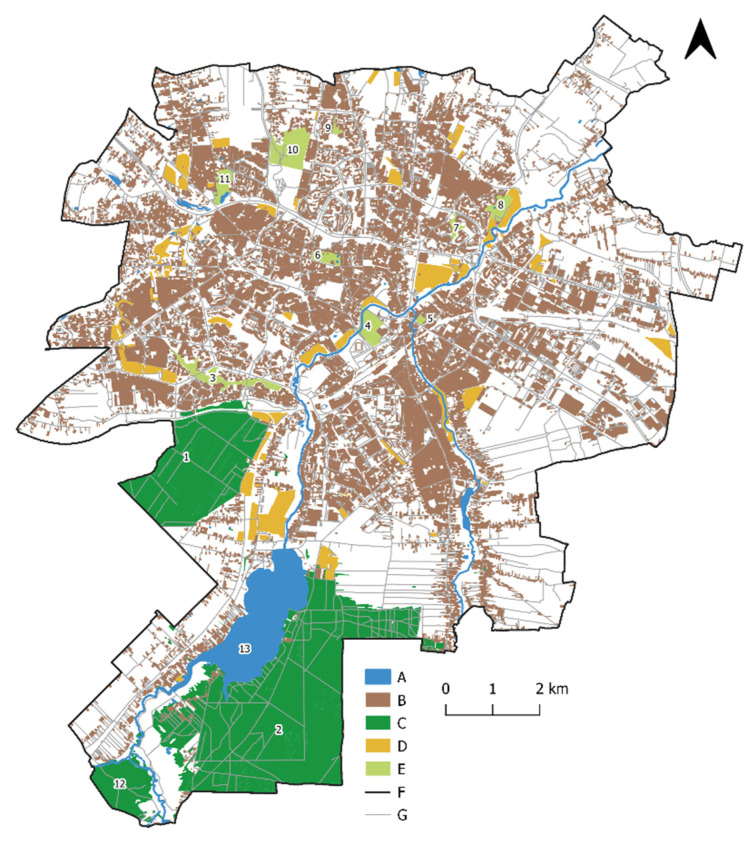
Selected recreation areas in Lublin. (A) surface water; (B) built-up areas; (C) woodland; (D) allotment gardens; (E) parks; (F) city boundary; (G) roads; (1) Stary Gaj forest; (2) Dąbrowa forest; (3) John Paul II Park; (4) Ludowy Park; (5) Bronowice Park; (6) Saxon Gardens; (7) Kalinowszczyzna gully; (8) Zawilcowa Park; (9) Czechow Park; (10) Górki Czechowskie Park; (11) MCSU Botanical Gardens; (12) Rudki forest; (13) Lake Zemborzycki (source: own elaboration based on [87,88]).

In 2020, green areas, defined as designed vegetation-covered areas (along with the associated buildings and technical infrastructure), account for 9.62% of Lublin’s total area, while parks, green squares, and green areas in housing estates account for 5.4% [85]. Lublin also boasts some woodland, several city parks (e.g., Saxon Gardens, Ludowy Park, Zawilcowa Park, John Paul II Park), and MCSU Botanical Gardens. There are also the so-called Family-Owned Allotment Gardens (e.g., ROD “Podzamcze”, ROD im. J. Czechowicza) (Figure 2).

## 4. Materials and Methods

The first case of SARS-CoV-2 infection in Poland was recorded on 4 March 2020: it was found in a person from Lubuskie Province, western Poland, from where the virus gradually spread to other regions, and eventually to all the provinces [89]. In the spring of 2020, numerous lockdown restrictions were introduced to stop the pandemic, which led to intensified symptoms of depression and anxiety. Universities were closed on the 11/12 March 2020, and remote learning was introduced [16].

The survey was conducted in the autumn of 2021 using a Google Forms online survey. A total of 381 students were surveyed; their participation was voluntary. The potential survey participants were informed that the survey results would be used for scientific research purposes. They were also assured of anonymity.

The first phase of the survey (October 2021) was concerned with the perception of landscape, while the second phase (November 2021)—with the importance of recreation areas before and during the pandemic. Separate specially prepared survey questionnaires were used in each phase. The first questionnaire covered the period from 11 March 2020 till 30 September 2021, and consisted of seven closed and open questions concerning, among others:

- the link between the selected place of recreation and the landscape.
- perceived changes in the perception of landscape and assessment of their relationship with the lockdown.
- rating the value of local landscape (related to the place of residence).
- proposals of changes in local landscape so that it provides a richer experience, conducive to recreation.

In the first phase of the survey, the respondents were divided into three groups representing students of Spatial Management, Tourism and Recreation, Geography, and other fields of study. The division was based on the differences in the competences of the students in the aforementioned courses, related to the varying educational profile. Tourism and Recreation’s students acquire general knowledge of natural, socio-economic sciences, protection and management of environmental resources and organisational and legal issues in tourism and recreation in an international context. Students of Spatial Management gain interdisciplinary knowledge of the organisation of space and the ways and factors of shaping it, especially in a national context. Furthermore, they are equipped with design and spatial planning skills (local and regional scale). As for geography students, they have a comprehensive knowledge of the various elements of the geographical environment and the mechanisms of their functioning. All these fields of study represent a spatial character and their students should be more or less aware of the meaning of landscape, its elements and its value.

The questionnaire used in the second phase was a continuation of the first phase. Due to slight differences in the answers received from specific groups of students in the first phase, the second phase was conducted without a division into groups. The questionnaire consisted of 18 closed, single, and multiple-choice questions (a few of them were constructed with the use of the 5-point Likert scale), and open questions, divided into two sections: before the pandemic (before March 2020) and during the pandemic (March 2020–September 2021). The questions concerned:

- an assessment of well-being.
- rating the influence of using recreation areas on improved well-being and activity.
- rating the need for contact with nature.
- rating the accessibility of recreation area near one’s place of residence.
- frequency of using recreation areas.
- types of recreation areas chosen for leisure activity.
- landscape features influence the choice of a place for recreation.
- recreational activities.
- the most frequented, specific places (a place name).

Either questionnaire also contained information on the gender, field of study, and place of residence of a respondent.

A total of 181 persons participated in the first phase of the survey, mainly students of Tourism and Recreation and Spatial Management. Most of the respondents indicated a village (77 persons) and a city of over 50,000 inhabitants (62 persons) as their place of residence. The respondents (29 persons) also lived in towns with a population of less than 50,000. Most of the respondents lived in Lubelskie Province. In the second phase, 200 students were surveyed, mainly students of Spatial Management. Among students of other faculties were students of Tourism and Recreation, Geography, Public Administration, Biocosmetology, Chemistry, Dietetics, Journalism and Public Communication, Economics, Physiotherapy, Military Geography and Crisis Management, Geoinformatics, German Studies, Computer Science, Environmental Engineering, Criminology, Medicine, Applied Linguistics, Speech Therapy with Audiology, Law, Russian Studies, and Sociology. Most of the respondents indicated a village (77 persons) and a city of over 100,000 inhabitants (62 persons) as their place of residence. The respondents also lived in towns with a population of less than 20,000 (28 persons), between 20,000 and 100,000 926 persons), and over 500,000 (7 persons). Most of the respondents lived in Lubelskie Province (Table 1).

The groups surveyed in either phase were not identical, but a considerable part of the respondents (about 60%) took part in both phases. Therefore, the results of the first phase and the second phase were not compared. The first phase was an introduction (context) to the more in-depth second phase of the research, from which results were statistically analysed. The findings of the second phase of the study were mainly considered in the conclusions. From the first phase, the most significant outcomes were suggested changes to the landscape to provide a more enriched experience conducive to recreation. Only the gender differences in responses were considered in the second phase. The field of study was not considered, based on the assumption that, although it may partly influence the responses, it was irrelevant to the purpose of the study. Similarly, the respondents’ place of residence was not considered.

The obtained survey results were exported from Google for analysis using the software Microsoft Excel and Statistica 13.3 version (TIBCO Software Inc., Palo Alto, CA, USA). As indicated above, the second phase was the more in-depth part of the research. Hence, to determine whether, in the second phase, statistically significant differences occurred between the perceived importance of recreation areas, as viewed by students from universities in Lublin, for the periods before and during the COVID-19 pandemic, the ANOVA Friedman test was used. The test was applied to the closed questions. Statistical significance was generally assumed at *p* < 0.05.

In the case of the open questions, the respondents’ answers were analysed using the text exploration technique [90]. The initial work consisted of filtering the text to reduce the number of irrelevant words and to combine words with the same meaning. Then the frequency of occurrence of words was calculated. In addition, content analysis [91] and thematic analysis were carried out [92]. It was also decided to select interesting statements enabling a better understanding of the respondents’ arguments. This analysis was carried out only for three open questions, two from the first phase (perceived changes in the perception of landscape and proposals of changes in local landscape so that it provides a richer experience, conducive to recreation) and one from the second phase (the most frequented, specific places). Although the computer software (CAQDAS, QDAS) could be helpful in this study, due to the relatively small study sample and the high cost of purchasing the software, it was decided to count repeated phrases or words in the texts by the authors themselves. Regarding longer statements, the characteristics of the information conveyed in the text were considered, and subsequently those with similar characteristics were grouped together and submitted to interpretation. Therefore, it was a qualitative study, using the methodology of this type of research, mainly used in the social sciences and allowing for advanced statistical elaboration in the future [93,94]. The results of the qualitative approach were presented mainly through a selection of interesting statements about perceived changes in landscape perceptions. For all open-ended questions, the qualitative approach was reinforced with a quantitative approach by counting the frequency of repeated responses. The resulting value was presented in the form of a percentage of all responses to a specific question.

## 5. Results

### 5.1. What Are the Students’ Preferred Landscapes for Recreation Purposes?

**Figure 3:**
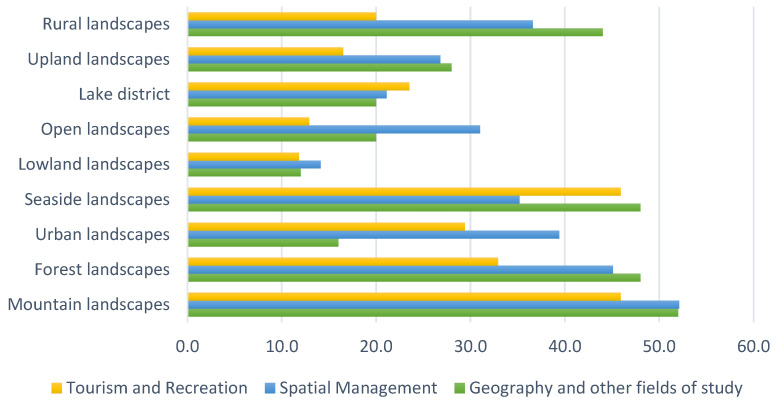
Landscape preferences among surveyed students (%).

The results of the first phase of the survey show that most of the Tourism and Recreation students surveyed (84.7%) had the possibility of going away from home to rest. For 82.4% of the respondents, the choice of a place of recreation was related to landscape. The respondents were choosing mountain and seaside landscapes as well as forest, urban, lake district and rural landscapes. Upland, open, and lowland landscapes were chosen less frequently (Figure 3). They were travelling mostly around Poland (43.5%) and other European countries (25.9%).

Most of the Spatial Management students surveyed (91.5%) had the possibility of going somewhere away from home for recreation. For 88.7% of the respondents, the choice of a place of recreation was related to landscape. The respondents chose mountain and forest landscapes as well as urban, rural, seaside, open, upland, lake district, and lowland landscapes (Figure 3). They travelled mostly around Poland (63.4%), while a minority travelled to other European countries (25.4%).

The vast majority (96%) of surveyed students of Geography and other fields of study had the possibility of going somewhere away from home for recreation. For the same respondents, the choice of the place of recreation was related to landscape. They chose mountain, seaside, forest and rural landscapes. Upland, open, lake district, urban and lowland landscapes were chosen less frequently (Figure 3). They travelled mostly around Poland (56%) as well as to other European countries (20%).

**Figure 4:**
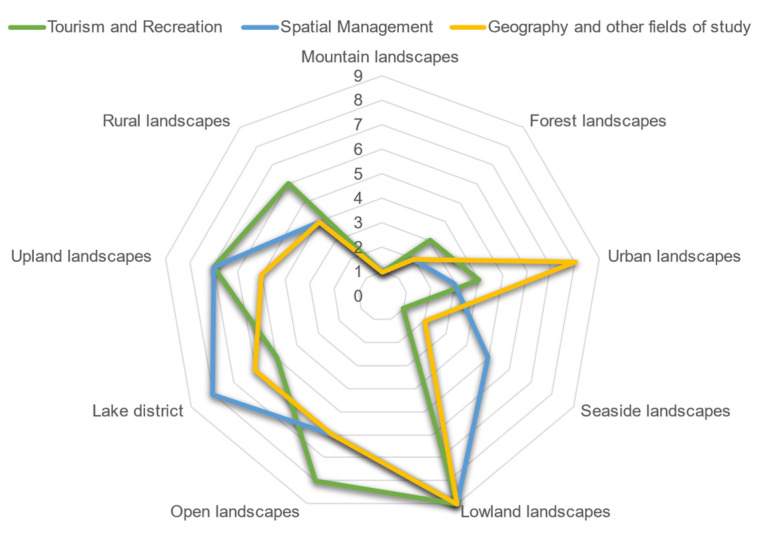
Landscape preferences. 1—The most frequently chosen landscapes; 9—the less frequently chosen landscapes.

Among all surveyed student groups, mountain and forest landscapes were the most frequently chosen landscapes. Lowland landscapes, on the other hand, were chosen the least frequently. Urban landscape was indicated more often by tourism and recreation students and spatial management students than by geography and other fields of study students (Figure 4).

### 5.2. Did the COVID-19 Pandemic Result in a Change in the Perception of Landscape?

**Figure 5:**
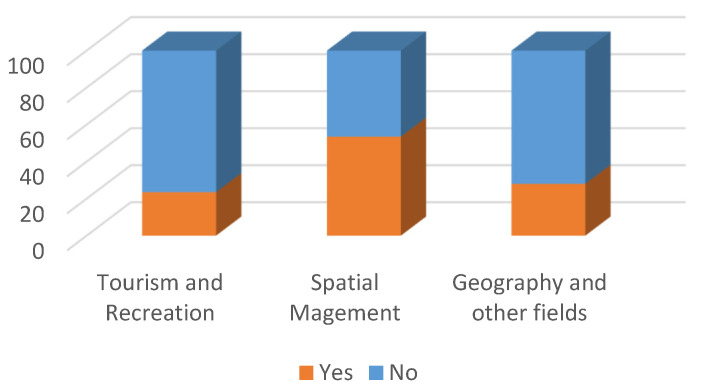
Survey question: Have you noticed a change in your perception of the landscape between 11 March 2020 and 30 September 2021? (%).

**Figure 6:**
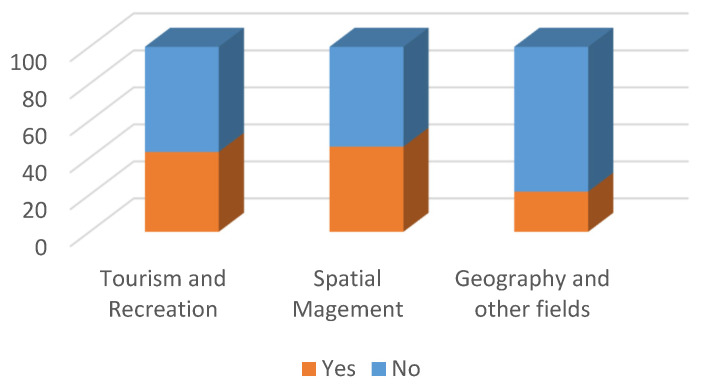
Survey question: Is the change in the perception of the landscape related to the “lockdown” resulting from the sanitary regime? (%).

Most of the Tourism and Recreation students surveyed did not notice a change in their perception of landscape (Figure 5). For the remainder, the change mostly consisted of appreciating the value of nature (59.4%), beauty and diversity of landscape as well as its healing and soothing effect (31.3%). Some 43.2% of the respondents indicated that the change had been related to the lockdown (Figure 6).

With respect to changes in the perception of landscape, the Spatial Management students surveyed were divided: 53.5% stated that they had noticed them while 46.5% stated they had not (Figure 5). The change mostly consisted of appreciating the value of nature (26.1%), beauty and diversity of landscape, including interesting views (63.0%). Below are some of the answers from the respondents:

- “After spending a long time in the comfort of your home and at the computer, going outside and admiring the landscape gave even more relief and a moment of relaxation than before, you could appreciate and enjoy it even more than previously, you could appreciate the brief time spent in nature as well as the various stimuli associated with it. Admiring the landscape made and still makes it possible to get away from reality for a while and forget about everything for a few moments.”
- “I started paying more attention to the urban landscape, how modern buildings blend in with historic buildings and whether they form an ideal urban landscape.”
- “I enjoyed the view of the landscape more, I appreciated the fresh air more, I paid more attention to the trees, the greenery.”
- “I pay more attention to ‘ordinary’, common areas. I notice more details in them, and they form a beautiful whole. I grew up in the countryside, and it’s only recently that I’ve become aware of agricultural, rural landscapes.”

The answers are thus quite encouraging, indicating a greater interest in the landscape, although there are also a few answers to the effect that nothing has changed. A total of 46% of the respondents noticed that the change had been related to the lockdown (Figure 6).

Most of the surveyed students of Geography and other fields of study did not notice a change in their perception of landscape (Figure 5). For the remainder, the change mostly consisted of appreciating the value of green areas and, more broadly, nature (77.8%). For the majority (78.3%), the change had not been related to the lockdown (Figure 6).

### 5.3. How to Design and Develop the Landscape to Provide a Richer Experience, Conducive to Relaxation?

The local landscape was of high or medium value for most of the Tourism and Recreation students surveyed (36.5% and 32.9%, respectively). The proposed changes in landscape so that it could offer a richer, more relaxing experience mostly consisted of introducing more greenery (46.3%) as well as reducing built-up areas and motor traffic (42.6%) and creating places with interesting views (11.1%).

The local landscape was of high value or medium value for most of the Spatial Management students surveyed (45.1% and 42.3% respectively). The proposed changes in landscape so that it could offer a richer, more relaxing experience mostly consisted of introducing more greenery/green areas conducive to recreation (37.5%) as well as improving the recreation infrastructure (walking lanes, arbours or gazebos, benches) (32.8%), creating viewpoints, removing advertisements and billboards that obstruct views (29.7%), etc.

The local landscape was of high value for most of the surveyed students of Geography and other fields of study (52%). The proposed changes in landscape so that it could offer a richer, more relaxing experience conducive to recreation mostly consisted of introducing more greenery and water (42.1%), as well as devoting more care to historic monuments, reducing concrete-covered spaces and motor vehicle traffic (42.1%), creating places with interesting views, regulating the placement of advertisements and announcements (leaflets, banners, etc.) in public space, and eliminating rubbish dumps (15.8%).

### 5.4. Were Recreation Areas Conducive to Improvement in Well-Being and Stress Reduction in Students during the COVID-19 Pandemic?

**Figure 7:**
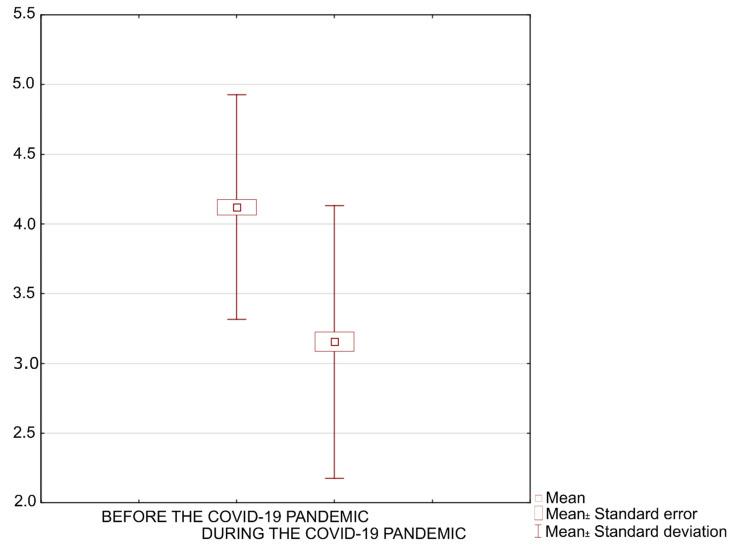
The respondents’ well-being before and during the COVID-19 pandemic.

The second phase of the survey enabled assessing the importance of changes before and during the COVID-19 pandemic, both in terms of the students’ well-being and the significance and perception of recreation areas. The Friedman Test shows that there was a statistically significant difference between young adults’ sense of well-being before and during the COVID-19 pandemic, X^2^_F(1)_ = 98,482, *p* < 0.001 (Figure 7).

According to the self-assessment at the start of the second phase of the survey, the respondents’ sense of well-being decreased. The percentage of respondents reporting moderate and poor well-being increased (by 26.5% and 14%, respectively). Notably fewer respondents reported good or very good well-being (decrease by 27% and 18.5%, respectively). The biggest changes were observed among students who had declared good or very good well-being before the pandemic began; their sense of well-being usually deteriorated to a medium level. Respondents who reported moderate or poor well-being before the pandemic experienced its deterioration to a lesser extent (Table 2). The survey results also indicate that women experienced the pandemic-related stress to a greater extent. Among women, an increase of moderate well-being (by ca. 30%) and decrease of good (by ca. 22%) and very good well-being (by ca. 30%) were the most conspicuous. Among men, the most noticeable change concerned very good well-being (decrease by ca. 19%) and moderate well-being (increase by ca. 15%).

According to the respondents, using recreation areas had either a very big (47%) or small (31%) influence on an improvement of well-being before the pandemic. These areas did not exert any influence on improved well-being for only 5% of the respondents. Recreation areas during the pandemic primarily contributed to an improvement in well-being and reduction of stress, as well as physical activity, functioning like the pre-pandemic times, and interpersonal contacts (Table 3).

**Figure 8:**
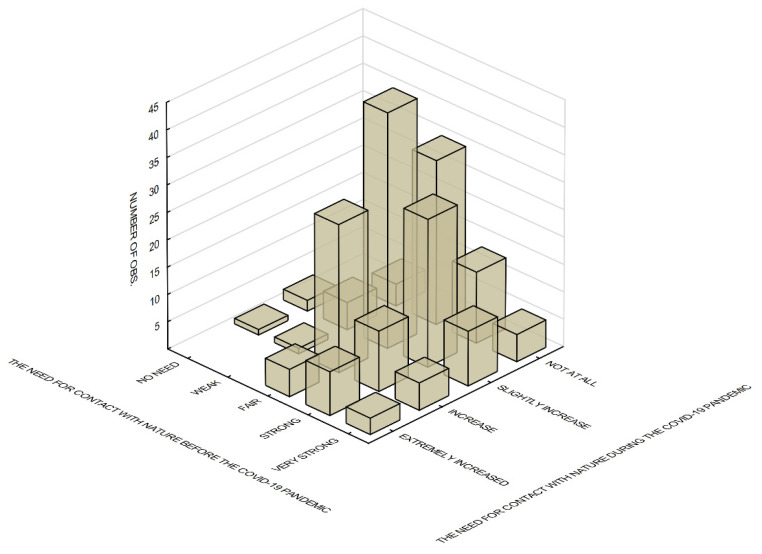
The need for contact with nature before and during the COVID-19 pandemic.

The Friedman Test shows that there was a statistically significant difference between the need for contact with nature before and during the COVID-19 pandemic, X_2F(1)_ = 126,025, *p* < 0.001. A moderate need for such contact was predominant before the pandemic (52.5% of the respondents); this need became slightly stronger during the pandemic. Respondents who felt a strong or very strong need for contact before the pandemic indicated a slight intensification of this need during the pandemic. The need remained unchanged in about one quarter of those surveyed (Figure 8).

### 5.5. Did the Opinions about the Accessibility of Recreation Areas Change during the COVID-19 Pandemic?

**Figure 9:**
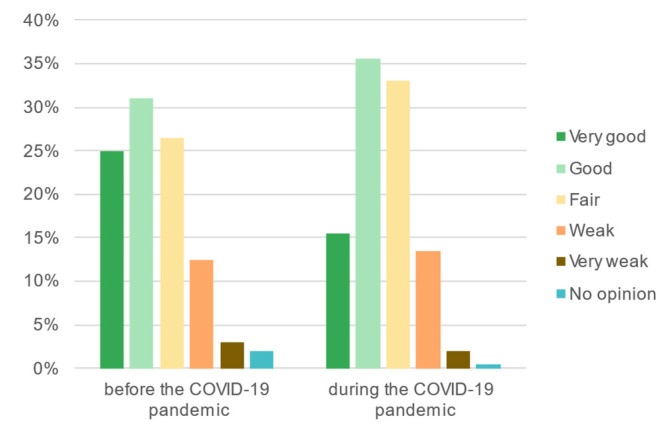
Rating the accessibility of recreation areas before and during the COVID-19 pandemic.

The difference in rating the accessibility of recreation areas before and during the COVID-19 pandemic was statistically significant at *p* = 0.031. A deterioration of accessibility, as viewed by the respondents, was visible. A large decline (by nearly 10%) was observed in the number of respondents rating accessibility as ‘very good’, whereas an increase occurred in the case of the ‘adequate’ and ‘good’ rating (by 6.5% and 4.5%, respectively) (Figure 9). The most frequent changes were by one level of rating: from ‘very good’ to ‘good’, and from ‘good’ to ‘adequate’. It is apparent then that during the lockdown, the respondents began to notice a shortage of recreation areas close to where they lived. This can result from the lower need for using such areas or a smaller amount of free time in the time before the pandemic.

### 5.6. Did the COVID-19 Pandemic Increase the Frequency of the Students’ Use of Recreation Areas and What Were the Recreational Activities Practiced?

The frequency of use did not change considerably. The Friedman Test shows that there was no statistically significant difference between the frequency of using recreation areas before and during the COVID-19 pandemic, X^2^_F(1)_ = 0.0099, *p* = 0.921. However, differences occurred in the answers with respect to the gender of the respondents. While women most often used recreation areas at weekends, men most often indicated that they used them a few times a week.

Most of the recreational activities in recreation areas did not change substantially. In both periods analysed, walking and meetings were predominant. The Friedman Test shows that there were statistically significant differences regarding the choice of cycling, roller-skating, meetings with friends/boyfriend/girlfriend (*p* < 0.05) and walking the dog (*p* < 0.1) before and during the COVID-19 pandemic. On the other hand, a decline in meetings (by 11.2%) was observed during the pandemic. Furthermore, fewer respondents chose cycling (decline by 10.7%) roller-skating (decline by 4.1%) during the pandemic. On the other hand, walking the dog was the most frequently chosen activity (increase by about 2.5%). Differences by gender are also evident here. During the pandemic, an increase in activities such as walking and walking the dog was greater among men than among women. Running was also more important for men than for women. Team games were prominent among other activities before the pandemic, but they were absent during the pandemic due to the lockdown.

### 5.7. What Types of Recreation Areas Were Chosen for Leisure Activity, What Land-Scape Features Influenced the Choice of a Place for Recreation and What Were the Most Frequented Specific Places?

The respondents indicated similar types of recreation areas chosen for leisure activity before and during the pandemic. There was a decline in interest in most types of areas, particularly water sites (lake, pond), accompanied by an increased interested in forests. The differences were not statistically significant for gullies, a botanical garden, allotment gardens, one’s own plot of land, and other recreation areas (Table 4). While gullies were less frequented by women during the pandemic, a slightly increased interest in these areas was visible among men. The parks were also less frequented.

**Figure 10:**
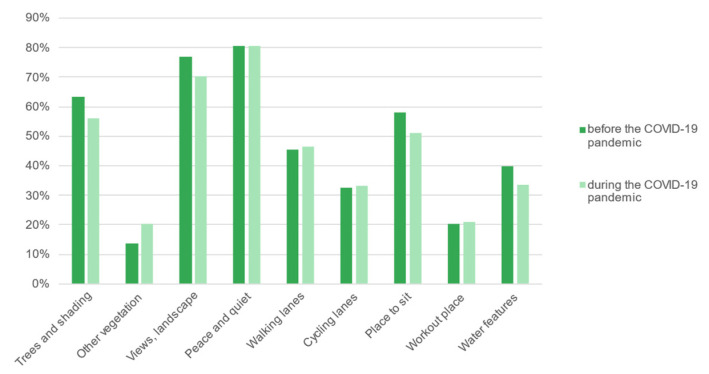
Landscape elements affecting the choice of recreation area before and during the COVID-19 pandemic.

The choice of a place of recreation, regardless of the period, was primarily influenced by the presence of tranquillity (peace and quiet), interesting views (landscape), trees and shading, places to sit, and walking lanes. The presence of trees, rest areas, and the surrounding views (*p* < 0.05) and water features (*p* < 0.1) had a smaller impact (decline by about 6–7%) on the respondents’ choices during the pandemic. However, the importance of other vegetation increased (*p* < 0.05). For the remaining elements (tranquillity, walking lanes, cycling lanes, workout places and other elements), the differences were not statistically significant (Figure 10). Interestingly, the trends among men were opposite to the above: although the differences between the time before and during the pandemic were small (about 1–1.5%), they attached greater importance to shading, landscape, and rest areas. The importance of other vegetation decreased for them, however.

Local or regional parks (both city parks, national and landscape parks) and forest were the most frequented. An increased importance of forests and decreased importance of cities, including green and blue infrastructure, were evident during the pandemic. Students indicated that, before the pandemic, they had visited tourist towns such as Kazimierz Dolny, Zwierzyniec czy Zamość. Zemborzycki Lake and nearby lakes (e.g., Lake Firlej or Lake Białe in the Łęczna-Włodawa Lake District) also used to be visited quite often. These places were less frequented during the pandemic. Out of all the sites mentioned in the questionnaire, the biggest number of the respondents indicated the Saxon Gardens in Lublin (a historic city park located close to the universities). This park was less frequented during the pandemic, which may have resulted largely from remote learning and the consequent absence of students in Lublin. However, interest in Lake Zemborzycki, Ludowy Park or the Botanical Gardens did not decrease to such an extent (Table 5). A greater diversity of parks visited during the pandemic could also be observed.

## 6. Discussion

For most of the respondents, the choice of the place of recreation was related to landscape. Stimulus-rich landscapes—mountain and seaside as well as forest landscapes—enjoyed the greatest popularity. The respondents travelled mostly around Poland. A considerable portion of the respondents did not notice any change in their perception of landscape. In the case of the remainder, the change mostly consisted of appreciating the value of nature, the beauty and diversity of landscape, including interesting views, as well as its therapeutic effect. For a considerable number of the respondents, this change was related to the lockdown.

The crisis during the COVID-19 pandemic influenced an increase in the importance of recreation areas for students from universities in Lublin. The results of the conducted analysis were consistent with other studies, e.g., [50] and confirm that during the COVID-19 pandemic, the respondents noticed an increased need for contact with nature. Although in some surveys, e.g., [7,50], it was shown that physical exercise was the main reason for visiting green areas, the survey conducted in Lublin, shows that recreation areas had a greater importance for students in the context of mental rather than physical health. Similarly, in Cracow, the outdoor gym was not so essential [67].

It was tranquillity cf. [50] and the surrounding landscape that were the most important features that influenced the choice of the place of recreation among students in Lublin during the pandemic. An increase in everyday activities, such as walking the dog, was evident in the Lublin survey. Although in Cracow the tendency was different (walking a pet was not the most important reason) [67], the increase was confirmed by Ugolini et al. [7], among others.

While a growing awareness of nature’s influence on mental health was observed, for many years it did not have a significant impact on planning [54]. The time of the pandemic was a challenge that paved the way for changes in the design/planning of space. The lockdown measures—restricting or simply preventing interpersonal contacts, freedom of movement, and freedom of choosing how to spend one’s leisure time—highlighted the special role of open spaces in maintaining the physical and mental health of city dwellers [95]. The strictly prohibited access to green areas resulted in increased interest in recreation outdoors after the lockdown [96]. Ensuring the possibility of using space in a safe manner became a key challenge for city administrations [97]. The pandemic triggered changes in the use of urban public spaces, and these processes will influence how open spaces will be designed and used in the future [12,73,98].

What is important in the case of green areas is their accessibility and proximity to one’s home, as well as their quality and development, including access to paths and lanes [13,27,68]. Bearing in mind the importance of green spaces in the context of residents’ health, our study and other studies suggest that it might be important to pay attention to the accessibility of these areas. There is no doubt that the distance to the nearest recreational area is important for its potential users [99]. The closer a green area is to a place of residence, the more likely it is visited by residents [100]. Similarly, our findings suggests that in the case of Lublin students changed the rating of the accessibility of recreational areas during the pandemic. The shortage of these areas in the vicinity of the home was noticed.

In urban planning and design, it might be important to consider the diversity of green areas, from large parks offering open spaces to pocket parks and gardens at a close distance to one’s home. Furthermore, it is necessary to connect green areas with a system of walking and cycling lanes that ensure green mobility [7]. Social distancing is a new health standard in urban design, alongside the recognition of active mobility based on walking and cycling, promotion of pedestrian traffic instead of car traffic [101].

Surveying the opinions of students on the importance of landscape and recreation areas can contribute to changes in the spatial policies of cities, particularly those having universities. The students surveyed indicated the need to introduce more greenery and reduce the amount of concrete in urban space. Similar recommendations follow from the research by Ugolini et al. [7]. Everyone has the right to landscape—the right to use the common assets perceived by the senses. Our study suggested that landscape and public space enabling recreation outdoors should hold an important place in the spatial policies of cities. The evident worse rating of the accessibility of recreation areas during the pandemic indicates the necessity to ensure pedestrian accessibility to increasingly vast green recreation areas both within city boundaries and in smaller localities [100]. Our study suggested paying more attention to highlighting valuable views and the presence of quiet areas that are part of the green and blue infrastructure in cities. This need is emphasised by the respondents’ motivation behind their choice of recreation areas during the pandemic. Other studies have indicated that the noise level in such areas should not exceed 55dB Lden, while their area should be at least 1 ha and within a 10-min walking distance [60].

More remote green public areas result in private gardens having a greater importance in the context of healthcare [27]. This indicates the key importance of public areas, particularly in cities where multi-family housing devoid of privately-owned plots of land predominates. This role can be played by city parks, especially those covering larger areas and not adjoined by sites or facilities causing nuisance [102]. As E.D. Ekkel and S. de Vries pointed out, a more sophisticated accessibility indicator to green areas may not be easy to apply in spatial planning, but would be more useful [99]. De Luca et al. applied a hierarchical approach to examine the accessibility of green spaces in Bologna, which took into account the class of the area and its size [103]. The larger the area, the greater its potential to attract visitors. The size can also be related to the class of recreational area [104]. Furthermore, the quality of the area and its aesthetic quality is also crucial [68,104]. Although proximity significantly influences the frequency of use of green spaces, residents are able to cover greater distances to the most attractive places, as confirmed by studies in Krakow [67] for instance.

Furthermore, protected areas contribute to well-being. The findings show that students in Lublin chose protected areas as a place of recreation relatively often. As students from Lublin also indicated the need for peace and quiet as one of their main motivations for visiting recreational areas, our results confirmed the research conducted by the Jiricka-Pürrer et al. They noticed that Polish young adults were likely to visit protected areas while seeking tranquillity [105].

The survey had some limitations resulting from the focus on university students in a Polish city. Expanding the survey to include students in other cities as well as members of other social groups, particularly senior citizens, is under consideration. In addition, there are plans to assess the level of activity in urban space and the level of satisfaction of its users, which will enable assessing the perception of the landscape of a given city [106] also based on its soundscape [107].

## 7. Conclusions

The most recent years, under the grip of the COVID-19 pandemic, have surely led to a deterioration of well-being in society, including young adults. Recreation areas facilitating, among other functions, interpersonal contacts, can help alleviate stress and improve the sense of well-being [7,8,9,10,15,77].

The findings suggest that the importance of recreation areas increased during the pandemic in the Lublin case study. The nature and therapeutic effect of the landscapes were appreciated more. The experience of the COVID-19 pandemic among students shows that it is advisable to provide residents with access to recreational areas. Our study suggested that it might be important to have a variety of green areas, reduce the size of built-up areas, and limit car traffic. In landscape design proposed to pay attention to the creation of places with interesting views, ensuring the removal of advertising and billboards that obstruct views, and providing appropriate recreational facilities (footpaths, gazebos, benches) is necessary.

The preparation of regulations for spatial planning and urban design, considering the availability of recreational areas within a 10 to 15-min walking distance, could improve the quality of life of residents. However, it is also important to consider the quality of the recreation area. Residents can cover greater distances to the places of high-quality landscape.

Further research is needed to study the landscape attributes of recreation areas conducive to recreation after the experience of the COVID-19 pandemic and the delimitation of quiet areas as a specific type of recreation area.

## Figures and Tables

**Table 1:** General characteristic of respondents in the surveys, 1st phase = 181, 2nd phase = 200.

Variable	Value of Variable	Total Number		Share of Respondents (%)	
		1st Phase	2nd Phase	1st Phase	2nd Phase
Gender	Females	118	147	65.2	73.5
	Males	63	53	34.8	26.5
Faculty	Spatial Management	71	85	39.2	42.5
	Tourism and Recreation	85	42	47.0	21.0
	Other faculties	25	73	13.8	36.5

**Table 2:** The change in the respondents’ sense of well-being before and during the COVID-19 pandemic.

		During the COVID-19 Pandemic					
		Very Bad	Poor	Moderate	Good	Very Good	*Total*
**Before the COVID-19 pandemic**	**Very bad**	0	0	1	0	0	*1*
	**Poor**	1	3	0	0	1	*5*
	**Moderate**	1	9	19	4	0	*33*
	**Good**	4	12	41	29	5	*91*
	**Very good**	5	9	25	21	10	*70*
	***Total***	*11*	*33*	*86*	*54*	*16*	*200*

**Table 3:** Rating the impact of recreation areas on health and activity level during the COVID-19 pandemic (%).

	Improvement in Well-Being	Reduction of Stress	Interpersonal Contacts	Physical Activity	Functioning Similar to the Pre-Pandemic Times
Totally agree	41.2	37.9	26.7	29.1	24.2
Agree	36.1	36.4	36.4	38.2	40.4
Disagree	5.7	10.6	13.3	11.1	15.2
Totally disagree	10.8	9.6	13.8	12.1	13.1
No opinion	6.2	9.6	9.7	9.5	7.1

**Table 4:** Types of recreation areas chosen for leisure activity (%).

	Before the COVID-19 Pandemic	During the COVID-19 Pandemic
Park **	66.8	57.7
Gullies ^N^	28.6	26.5
Forest ***	68.4	72.4
Lake/pond *	63.8	49.0
Botanical garden ^N^	14.3	11.2
Allotment gardens ^N^	19.4	18.4
Own plot of land ^N^	52.6	50.5
Other ^N^	4.1	3.1

The differences are statistically significant at * *p* < 0.001; ** *p* < 0.05; *** *p* < 0.1; ^N^—there was no statistically significant difference.

**Table 5:** The most visited places (%).

	Before the COVID-19 Pandemic	During the COVID-19 Pandemic
Protected areas	13.5	12.0
Forests	24.0	28.0
Saxon Garden (Lublin)	26.5	17.0
Other city parks and gullies	28.5	29.0
Zemborzycki Lake (Lublin)	11.5	9.5
Other lakes	13.0	12.5
Tourist towns	9.5	4.0

## Data Availability

Not applicable.
